# Ski Position during the Flight and Landing Preparation Phases in Ski Jumping Detected with Inertial Sensors

**DOI:** 10.3390/s19112575

**Published:** 2019-06-06

**Authors:** Veronica Bessone, Johannes Petrat, Ansgar Schwirtz

**Affiliations:** 1 Department of Biomechanics in Sports, Faculty of Sport and Health Sciences, Technical University of Munich, 80992 Munich, Germany; johannes.petrat@tum.de (J.P.); ansgar.schwirtz@tum.de (A.S.); 2 Olympic Training Center of Bavaria, 80809 Munich, Germany

**Keywords:** kinematics, kinetics, injury prevention, performance, feedback, ski movements, landing, impact, telemark

## Abstract

Ski movement plays an important role during landing preparation, as well as in the whole ski jumping performance. Good landing preparation timing and correct ski position increase the jump length and reduce the impact forces. Inertial motion units (IMUs) placed on the skis could constitute a promising technology for analyzing the ski movements during training. During regular summer trainings, 10 elite athletes (17 ± 1 years) performed jumps while wearing IMUs and wireless force insoles. This set-up enabled the analysis of a possible correlation between ski movements and ground reaction force (GRF) during landing impact. The results showed that the pitch during the landing preparation is the most influential movement on the impact kinetic variables since it is related to the angle of attack, which affects the aerodynamics. The ski position at 0.16 s before landing did not influence the kinetics because the athlete was too close to the ground. During the impact, the roll angle did not correlate with GRF. Moreover, each athlete showed a different movement pattern during the flight phase. Concluding, the combination of IMUs and force insoles is a promising set-up to analyze ski jumping performance thanks to the fast placement, low weight, and high reliability.

## 1. Introduction

During ski jump landing preparation, as well as during the entire flight phase, ski position plays an important role in performance and safety. The ski position during landing preparation has been shown to increase the jump length by up to three meters [1]. In fact, a larger angle of attack (i.e., the angle between the ski and the air stream) enables the ski jumper to exploit the aerodynamic lift force and its cushioning effect. This effect permits the athlete to decelerate, with a consequent reduction of the impact forces [1] and, consequently, of the injury risk [2]. Delaying the landing preparation time has been demonstrated to be one of the performance factors, together with an effective take-off, a high initial velocity, and an efficient flying technique [3], and what distinguishes the high-ranked jumpers from the low-ranked ones [4]. The start and duration of the landing preparation have not been defined yet, but major differences of the ankle and hip angles were observed at 0.4 s before the landing impact, while knee joint variations were found at 0.14 s before the landing [4]. Moreover, during competitions, the landing technique is evaluated according to the Competition Rules of the International Ski Federation [5], and constitutes part of the points of the final score, together with jump length, wind factor, flight technique, and starting gate [5]. In particular, the athlete should land using the so-called “telemark”, a step landing position, difficult to perform but biomechanically more advantageous than the parallel leg landing (i.e., landing with the feet at the same height in a squat position) [2]. As a consequence, a correct ski positioning and timing of the start of the landing preparation permit the athlete to execute a correct telemark position, as well as reduce the impact force acting on the lower limbs [2].

During the flight phase, having a stable ski position is essential for performance and safety [3,6,7]. The ski jumper usually tries to keep a V-style ski position, since it has been shown to be more effective than the parallel position [6]. To achieve aerodynamic efficiency and stability, the athlete needs to continuously adjust his/her ski movements in order to compensate for external factors (such as the change of pressure and wind) that are acting on him/her, finding a compromise between a steady position and angular adjustments [7]. 

Consequently, the goal of the present study was to investigate the ski position during the ski jumping performance due to the aforementioned important role played during the flight and the landing preparation phases. The detection of the ski orientation could support trainers and athletes in improving technique and performance. The ski opening angle and the movement regulating the angle of attack have been determined using 3D video analysis. However, the rotation around the longitudinal axis of the ski (roll), responsible for the tilting movement, appeared to be inaccurate using video cameras, due to the difficulties of visually determining the ski rotation [8]. Compared to the 3D video analysis, the use of wearable sensors, such as inertial motion units (IMUs), could constitute an interesting set-up for in-field ski movement analysis, being able to detect the orientation of the skis more accurately.

IMUs placed on skis have been applied in previous studies dealing with skiing sports, in particular, cross-country (XC) skiing, ski mountaineering, and ski jumping. In XC skiing [9], a fixed sensor on the ski has been used to determined cycle duration, speed, and distance. In addition to these variables, skin off and on, kick-turns, slope angle, and elevation gain have been detected by IMUs in ski mountaineering [10,11]. With or without further sensors on body segments, IMUs placed on the skis have been used to detect the sub-techniques of classic [12,13] and skating [12,14] XC skiing techniques. Moreover, IMUs on the skis have been used to analyze the friction between skis and snow [15]. Finally, in ski jumping, inertial sensors have been employed by different authors, being light and with a wide recording volume [16], two important characteristics of wearable sensors for their use in this sport. Previous publications carried out data collections using IMUs on skis and on body segments in order to analyze the overall performance [17,18,19], or the take-off and in-run [20], or the lower body kinematics during the landing impact [21], but without deeply concentrating on the ski angular movement. The potential of the use of inertial sensors placed only on the skis to detect their position has been introduced and tested on one subject by Kreibich and colleagues [8]. Always with the sensors placed on the skis, Groh and colleagues [22] were able to detect the ski speed and the jump length. Moreover, the same author introduced the use of inertial sensors on the skis to detect the angular momentum during landing, validating it with custom-made force-measuring bindings [23].

To the best of our knowledge, no studies investigated the skis’ movement during landing preparation and the possible correlations with the landing kinetics, important factors for injury prevention and performance improvement. Therefore, a combination of IMUs placed on the skis and wireless force insoles could represent an interesting set-up for this analysis. This combination has been previously introduced by the authors of the present paper, and the first results showed that the ski position influences the vertical ground reaction force (GRF) [24]. The combination of IMUs and force insoles proposed in [24] was utilized in the present study on a higher number of ski jumpers to detect possible correlation between ski position and impact kinetics. Moreover, an overview of the ski movements during the flight phase was presented. 

The goal of the study was to achieve greater insight into the ski position during the flight phase by means of inertial sensors, with a particular focus on the landing preparation in order to detect correlations with the impact kinetics. We hypothesize that:

(i) Each athlete owns his specific ski pattern during the flight performance, depending on the competition level and expertise [7];
(ii) The pitch (rotation around the frontal axis) during the landing preparation is the ski movement that mainly acts on the impact kinetics, being related to the angle of attack [1];
(iii) The roll (rotation around the sagittal axis) during the impact influences GRF, since it influences the direction of GRF resultant vector;
(iv) Around 0.40 s before the landing impact as in [4], the main ski movements that lead to the start of the landing preparation happen.

## 2. Materials and Methods

### 2.1. Set-Up Description

Ten male ski jumpers competing at the National and International Junior level (17 ± 1 years) performed the test voluntarily during regular summer training conditions. Within the summer training preparation, the athletes perform on the regular ski jumping hill, while gliding in the in-run on watered ceramic tracks and landing on synthetic grass. Six athletes jumped on the ski jumping hill HS100 in Oberhof (Germany) and four athletes on the hill HS98 in Ramsau-am-Dachstein (Austria), during different days of data collection. Each jumper performed three jumps and landed using telemark or parallel leg landings, depending on jump length, wind conditions, and his expertise. The participants were verbally informed in full about the nature of the study and they were allowed to withdraw at any point without giving a reason. The protocol used in the study obtained the ethical approval from a designated Commission of the Faculty of Sport and Health Sciences of the Technical University of Munich.

**Figure 1:**
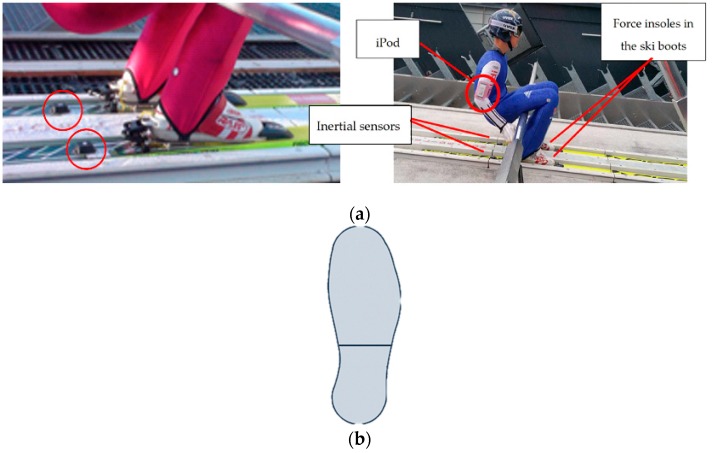
(**a**) Placement of the iPod case, force insoles, and inertial sensors on a subject; (**b**) Detecting area division of the fore and rear foot part in loadsol force insole (adapted from [29]).

The athletes jumped wearing force insoles and IMUs, with an overall weight of 0.3 kg. Both the wearable sensors were able to stop automatically after a predetermined period of time. The loadsol plantar force insoles (Novel GmbH, Munich, Germany) detected the normal GRF during landing, sampling at a rate of 100 Hz. The insoles were connected via Bluetooth to the app loadsol (Novel GmbH, Munich, Germany) installed on an iPod (Apple, Cupertino, CA, USA). The device worked as a data logger and was positioned on the arm of the athlete with a smartphone running case (Figure 1a). The force insoles have been previously validated [25,26,27,28] and detected the normal overall force (i.e., the force between the plantar side of the foot and the shoe) (Figure 1b) [29]. Before each jump, the force insole system was calibrated with the athlete’s body weight (BW) measured before the training using a body scale and including ski boots, helmet, gloves, and ski suit. During the calibration of the insoles, the athlete had to stand firstly on one foot, raising the other, and then vice versa. The values, collected by the force insole of the raised foot, were zeroed considering no forces acting on the foot.

Two IMUs (MSR Solutions, Wangen/Allgäu, Germany) were placed 0.1 m behind the binding and fixed with tape, one on each ski (Figure 1a). The sensors had a sample rate of 100 Hz and were composed by an accelerometer (±8 g), a gyroscope (±2000 °/s), and a magnetometer (±8 G). An operator activated the inertial sensors by Bluetooth using a laptop while the participant was sitting on the bench of the in-run. The IMUs stored the data on their internal memory.

### 2.2. Data Processing and Variable Definition

The data processing was conducted using custom-written Matlab 2017a (Mathworks Inc., Natwick, MA, USA) scripts. 

**Figure 2:**
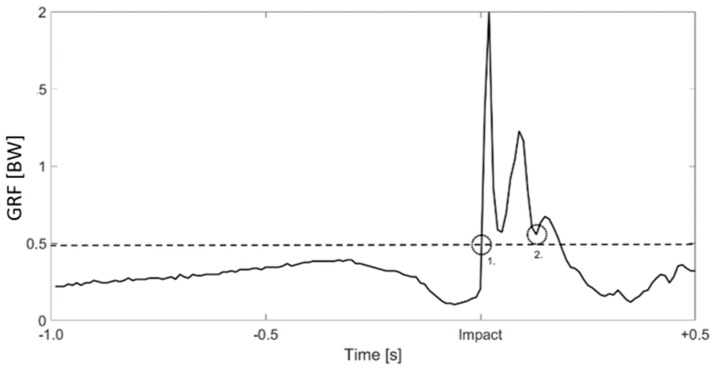
Normal ground reaction force (GRF) outcomes from one side, with the start (1.) and the end (2.) of the landing impact. The dashed line represents the 0.5 body weight (BW) threshold.

The normal overall GRFs recorded by each insole were normalized to BW and used as outcomes from the loadsol app, where the values are rounded by the app in steps of 5 N. The GRF_max_ was the maximal normal ground reaction force collected during the landing impact. The impulse I (1) was defined as

(1) $$I=\int_{t_{s}}^{t_{f}}GRF\;dt$$

where, as reported by [23], the start of the landing impact *t_s_* was defined as the first increase of the normal GRF (i.e., when GRF was higher than 0.5 BW). *t_f_* coincided with the minimum of the signal after the second GRF peak after touchdown, corresponding with the end of the eccentric phase (Figure 2) [23]. The difference between *t_f_* and *t_s_* defined the landing time (*t_landing_*). 

**Figure 3:**
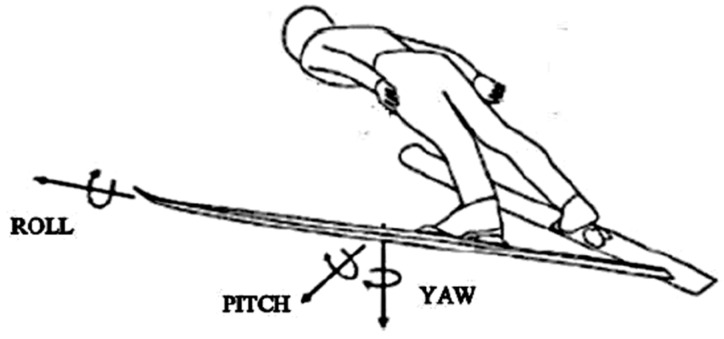
Representation of the roll, pitch, and yaw ski angles (adapted from [7]). The rotation arrows indicate how the positive directions of the ski angular movements were defined.

The raw IMU measurement data were postprocessed by using the algorithm presented in [30]. Firstly, the outcomes were low-pass filtered with cut-off frequency of 5 Hz. Subsequently, the algorithm reconstructed the attitude angles of skis offline by fusing gyroscope and magnetometer measurements with the geometric shape of the in-run [31], through an extended Rauch–Tung–Striebel smoother and maximum-likelihood principle-based state and parameter estimation algorithm. Therefore, the roll and yaw were set at 0° at the end of the in-run, considering the ski gliding flat and parallel in the tracks during this phase, while the pitch was set at −11°, according to the incline of the table reported in the design certificates of both ski jumping hills [31]. The reconstructed Euler angles of roll, pitch, and yaw were defined respectively as rotation around the longitudinal, frontal, and vertical axis of the skis (Figure 3) [9]. Thanks to this algorithm, before the start of the data collection, the calibration of the sensors was not necessary.

The roll angle of the left ski was defined positive when internally rotated, and negative for the right ski. The ski flexion defined a positive pitch for both the left and the right ski. The right ski opening angle was defined as positive when the tip of the ski was rotated in a clockwise direction, while for the left ski, when rotated anticlockwise. For analyzing the landing preparation, we calculated the ski angular range of motion (ROM) between ski angle during landing impact *t_s_* (set as reference) and at 1.00 s (*t_1.00_*), 0.76 s (*t_0.76_*), 0.56 s (*t_0.56_*), 0.36 s (*t_0.36_*), and 0.16 s (*t_0.16_*) before *t_s_*. Except for the 1.00 s before landing, the other time points were chosen according to a previous publication [4], in order to have the possibility of comparing the results on the base of the same time points. Due to the importance of the ski pitch during the landing preparation as in all of the flying phase, its differences (Δ) in the last second (1.00 s) before the landing were calculated between *t_1.00_*–*t_0.76_*, *t_0.76_*–*t_0.56_*, *t_0.56_*–*t_0.36_*, and *t_0.36_*–*t_0.16_* in order to determine when the main movement variations for preparing the landing are performed. The difference between *t_0.16_* and *t_s_* was not calculated as this period is too close to the impact.

For describing the ski movements during the flying phase, for each athlete, the ski angles of the three jumps were normalized to 100 samples, averaged, and visualized for each athlete separately.

The flying time (*t_flight_*) was calculated using the insoles and defined as the time between the end of the take-off and *t_s_*. The end of the take-off was defined when the normal GRF recorded by the insoles was below 0.5 BW after the in-run. The flying times calculated for the left and right side were averaged to obtain *t_flight_*_._ Due to the lack of video analysis around the landing area, *t_flight_* was utilized to determine the jump performance, assuming that a longer *t_flight_* corresponds to a longer jump length in comparable environmental conditions (same wind and weather) [21].

### 2.3. Statistical Analysis

The mean and standard deviation (SD) are reported in order to show the magnitude of the detected variables. The data collected on the two ski jumping hills were analyzed separately, due to the difference of the ski jumping hill design and the environmental conditions (such as different air pressure and wind). The kinematic and kinetic data were specified when related to the left (L) or right (R) side. The threshold for statistical significance was set at *p* < 0.05. To determine relationships between the kinematic and kinetic variables, Pearson correlations were calculated, considering each jump as a single case, even when performed by the same athlete. Paired sample t-test was applied to detect variations among the pitch movement differences during the landing preparation. The statistical analysis was performed using IBM SPSS Statistics (IBM Corp., Armonk, NY, USA). 

## 3. Results

### 3.1. Ski Movement during the Flight Phase

**Figure 4:**
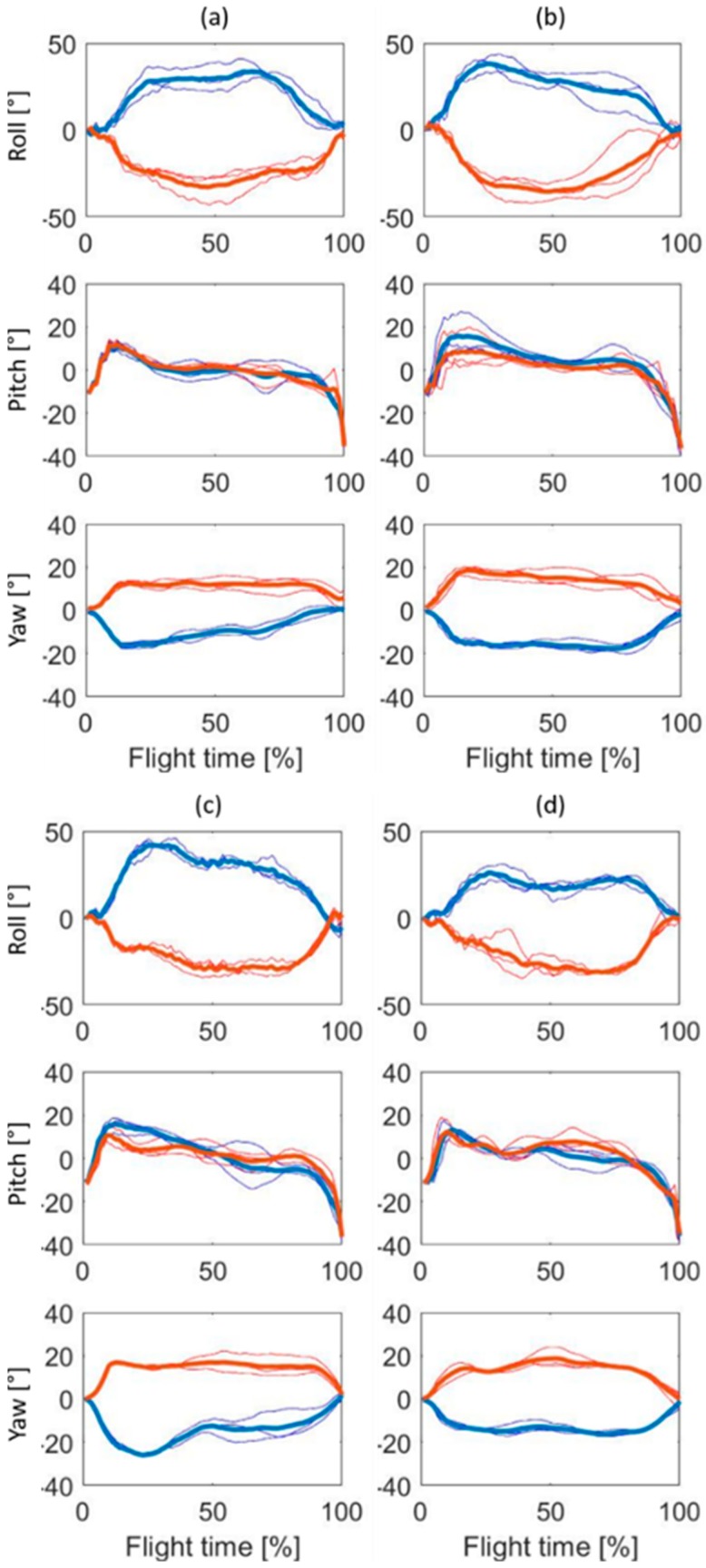
Normalized ski angular movements of roll, pitch, and yaw for three jumps of the four athletes collected in Ramsau-am-Dachstein (**a**–**d**). The blue line represents the left ski, the red line represents the right ski. The thick lines represent the average of the three normalized trajectories of each athlete, while the thin lines show the trajectory of a single jump.

**Figure 5:**
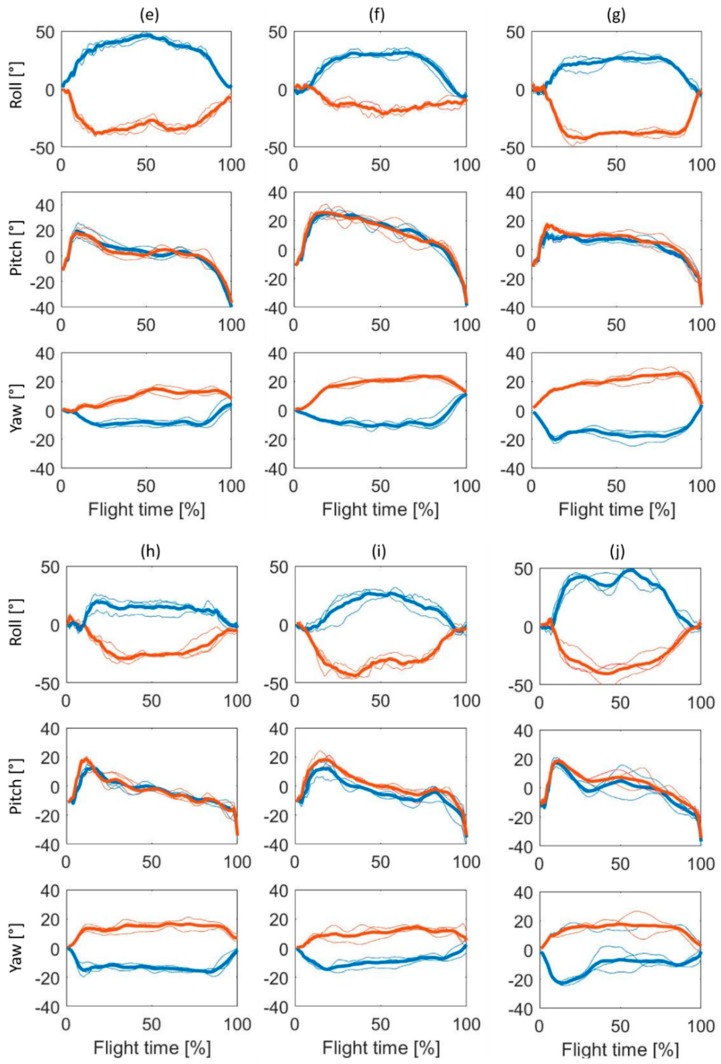
Normalized ski angular movements of roll, pitch, and yaw for three jumps of the six athletes (**e**–**j**) collected in Oberhof. The blue line represents the left ski, the red line represents the right ski. The thick lines represent the average of the three normalized trajectories of each athlete, while the thin lines show the trajectory of a single jump.

For visualization, three normalized ski angular movements of roll, pitch, and yaw during the flying phase (from end of take-off until the landing impact) collected for each of the 10 athletes are reported in Figure 4 and Figure 5 (jumps recorded in Ramsau-am-Dachstein: **a**–**d**, and in Oberhof: **e**–**j**, respectively), after being normalized on 100 samples and averaged.

### 3.2. Ski Movement during the Landing and Its Preparation Phases and Influence with the Kinetics 

The average *t_flight_*_,_ *t_landing_*, normal GRF_max_, and impulse I are reported in Table 1. Longer *t_flight_* did correspond to higher normal ground reaction forces (on the L foot: *r* = 0.774, *p* = 0.003; on the R foot: *r* = 0.580, *p* = 0.048 in Ramsau-am-Dachstein; on the L foot: *r* = 0.729, *p* = 0.001; on the R foot: *r* = 0.519, *p* = 0.027 in Oberhof). 

The normal GRF_max_ did not correlate with any of the ski angular movements at *t_s_* (landing impact) in Ramsau, but correlated with the pitch in Oberhof (GRF_max__L vs. L ski pitch: *r* = 0.610, *p* = 0.007; vs. R ski pitch: *r* = 0.581, *p* = 0.011; GRF_max__R vs. L ski pitch: *r* = 0.590, *p* = 0.010; vs. R ski pitch: *r* = 0.585, *p* = 0.011). On the other hand, *t_landing_* did not correlate with any ski movements at *t_s_* in Oberhof, while it did correlate in Ramsau. *t_landing_*_R correlated with the roll (*r* = −0.628, *p* = 0.029) and yaw (*r* = 0.606, *p* = 0.037) of the right ski. 

The correlations between the ski angular ROM at *t_1.00_*, *t_0.76_*, *t_0.56_*_,_ *t_0.36_*, and *t_0.16_* and *t_flight_* and the kinetic variables *t_landing_*, normal GRF_max_, and I of the data collected on the two ski jumping hills are shown in Table 2 and Table 3. For clarity, only the correlations where statistical significance was found are shown. Since no correlations were found with the ROM at t_0.16_ in Ramsau (*p* > 0.05), the data were not reported. 

**Figure 6:**
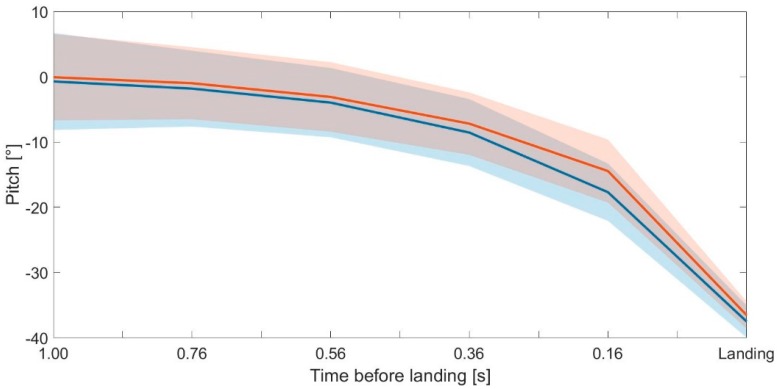
Average pitch movement and its SD from 1.00 before the landing impact until the landing for the left (in blue) and right (in red) ski at *t_1.00_*, *t_0.76_*, *t_0.56_*, *t_0.36_*, *t_0.16_*, and *t_0.00_* (landing impact) for the 30 collected jumps. A pitch angle of 0° corresponded to a horizontal/flat position of the ski in the air. Negative values corresponded to a movement of the ski tips in a clockwise direction (pointing down).

The average pitch movements and SD (*n* = 30) of the left and right skis from 1.00 s until the landing are reported in Figure 6. 

**Figure 7:**
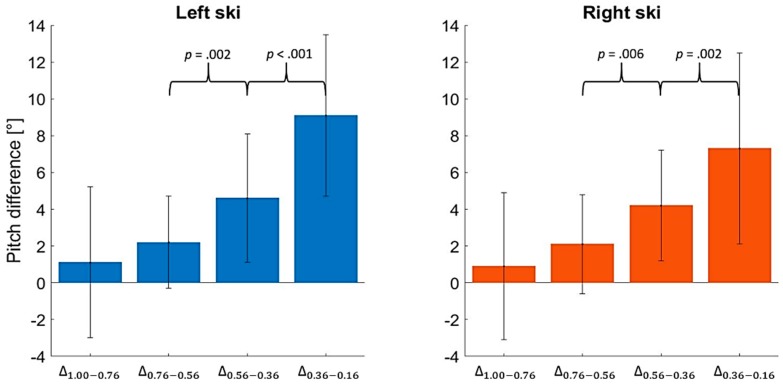
Difference Δ of the pitch movement between *t_1.00_*–*t_0.76_*, *t_0.76_*–*t_0.56_*, *t_0.56_*–*t_0.36_*, and *t_0.36_*–*t_0.16_* for the 30 collected jumps for the left and the right ski. The *p*-values indicate the statistical difference between the variations of contiguous ranges.

The pitch difference Δ between *t_1.00_*–*t_0.76_*, *t_0.76_*–*t_0.56_*, *t_0.56_*–*t_0.36_*, and *t_0.36_*–*t_0.16_* for the left and right skis are reported in Figure 7 with the related statistics. The statistical difference was calculated only between contiguous ranges (i.e., *t_1.00_*–*t_0.76_* and *t_0.76_*–*t_0.56_*, *t_0.76_*–*t_0.56_* and *t_0.56_*–*t_0.36_*_,_ and *t_0.36_*–*t_0.16_*).

## 4. Discussion

Referring to our hypotheses, the results showed that: (i) each athlete owns his specific ski pattern during the flight phase; (ii) the pitch during the landing preparation is the ski movement that mainly acts on the impact kinetics; and (iv) significant ski pitch variations happened between *t_0.36_* and *t_0.16_*, leading to the consideration that around 0.36 s before the landing there is the start of the landing preparation. However, different from the hypothesis (iii), the roll during t_s_ did not correlate with any of the kinetic variables during landing impact. 

### 4.1. Ski Movement during the Flight Phase 

As visually notable from the reported cases (Figure 4 and Figure 5), the curves of the ski angles are distinctive among the participants, owning their personal movement patterns depending on the expertise of the athlete [7].

During the phase of the first flight (i.e., the transition phase between the end of the take-off and the start of stable flight), the athlete needs to open the ski in a V-shape to rotate the ski internally and to raise the ski tips in order to increase the aerodynamic force acting on himself (Figure 4 and Figure 5). Each ski jumper attained stable flight in a different way. For example, subjects **a**, **d**, **e**, **f**, and **j** had a steep and symmetric pitch angle in comparison to the other subjects during the first flight (from 0% to 20% circa of the flight time in Figure 4 and Figure 5). After the first flight phase, some athletes kept a wide angle of attack (corresponding to a high angular value of ski pitch), reducing smoothly the angle during the flight phase (as **b** and **f**). Other athletes brusquely moved the ski in a horizontal position (around 0°), as athlete **e** (Figure 5). Moreover, some athletes (**c** in Figure 4 and **j** in Figure 5) showed an asymmetrical yaw angle.

During the flight phase (between 20% and 90% circa of the flight time in Figure 4 and Figure 5), the athlete should keep a stable and symmetrical position [5,6,7]. Referring to Figure 4 and Figure 5, it is possible to notice how some athletes kept an unstable position, with a lot of adjustment during the flight phase, as, for instance, subjects **f**, **h**, and **j** (Figure 5). At the same time, subjects **f** and **h,** together with subject **i**, never kept the same pitch angle during the flight, decreasing it constantly during the entire phase (Figure 5). The ski opening angle ranged between 30° and 40°, as previously reported to be the most efficient angular position [7,8]. The roll angle differed among athletes: Some participants kept the ski rotated around 50° (as **e** and **j**, Figure 5), while athlete **h** maintained relatively flat skis during the flight phase. A flat V-style has been shown to have better aerodynamic characteristics in comparison to a V-style where the skis are not so close to the body [32]. 

During the landing preparation (at the end of the flight phase, from 90% until the end of the flight time circa in Figure 4 and Figure 5), subject **a** changed the pitch angle with rapid movements, while subjects **b**, **c**, and **d** prepared the landing in a smoother way, changing the ski pitch slower (Figure 4). The athletes demonstrated having generally asymmetrical ski movements, independently of the roll, pitch, or yaw angle. The asymmetry could be explained by the expertise of the athlete [7], but also by the inconstant lift and drag forces acting during the flight that, differently from wind tunnel testing, cannot be excluded during in-field tests [8]. 

Considering as criteria for judging the quality of the ski position technique the previously mentioned statement that the athlete should keep a stable and symmetrical position during the flight [5,6,7], none of the athletes of the study showed an outstanding ski position technique. This could be explained by the fact that the participants of the study, despite being elite athlete members of the German National Team, belonged to the Junior category, in which a technical maturity is still not reached.

The representations in Figure 4 and Figure 5 are based on the normalization of ski movement data during the aerial phase of the ski jumping performance (from the end of the take-off until the landing impact). However, if the first flight and landing preparation time have a comparable duration among jumps of the same athletes, the flight phase has a different duration. This means that with a normalization to 100 samples, the data can be “stretched” or “compressed”, and therefore, potentially influence the visual representation. However, for each athlete, the three *t_flight_* were comparable in duration; on average, in fact, the difference among the jumps was of 0.10 s (3% of the average *t_flight_*). 

Finally, the analysis of the ski movement pattern is important during daily training of the athletes, and the use of inertial sensors could replace video cameras, providing reliable data without needing a lot of time for postprocessing or placing the cameras around the ski jumping hill.

### 4.2. Ski Movement during the Landing and Its Preparation Phases and Influence with the Kinetics 

The pitch was the main ski movement to correlate with *t_flight_* (Table 2 and Table 3), confirming the role of this movement during the flight phase, given its relation with the angle of attack and the consequent influence on the aerodynamic forces. In particular, wider ranges of motion of the pitch corresponded to longer *t_flight_* and consequently longer jumps. This means that the wider the difference between the ski pitch at landing and during the flight, the longer the jump length reached by the athlete. As a consequence, since the ski jumper needs to keep the skis flexed as long as possible in order to profit from the aerodynamic lift force [1], the athlete has to perform the landing preparation in a short time. No correlations were found between the pitch ROM and *t_flight_* at *t_0.16_*_._ This could be related to the fact that the athletes are close to the ground of the landing area at 0.16 s before the impact. Consequently, the angle of attack, controlled by the pitch movement, cannot influence the aerodynamic forces when the athlete is too close to the ground [1].

The magnitudes of the collected kinetic variables were comparable with the ones of previous publications [21,24,33] (Table 1). The correlation between the normal GRF_max_ and the ROM of the pitch before the landing (landing preparation) confirmed that the ski movements during this phase play an important role not only for the jumping performance, but also for safety, acting on the aerodynamic lift forces and their cushioning effect, reducing the impact forces [1] and, consequently, the injury risk [2] (Table 2 and Table 3). In particular, wider ROM of the pitch corresponded to smaller normal GRF_max_ (and impulse), while the roll and yaw did not have any correlations with the kinetic variables. 

Some of the collected kinetic variables correlated with certain kinematic variables differently among the ski jumping hills. For example, the impulse and normal GRF_max_ acting on the left side correlated with many kinematic variables collected in Oberhof (Table 1), but not in Ramsau-am-Dachstein (Table 2). This could be related to the fact that different athletes carried out the data collection on the two ski jumping hills. Consequently, their personal ski movement pattern could have influenced the kinetics in a different way. Therefore, a deeper analysis of the ski position pattern, as the one previously proposed, could give further information about the relation between ski movements and landing kinetics. 

Focusing on the ski pitch between t_1.00_ and t_landing_ (Figure 6), it is possible to notice objectively how between 1.00 s and 0.56 s before the landing, the athletes kept a stable position. In fact, the average differences between the ski position of the left and right ski in the ranges *t_1.00_*–*t_0.76_* and *t_0.76_*–*t_0.56_* were of 1.0° ± 4.1° and 2.1° ± 2.6°, respectively. Moreover, no significant difference was found between the variations *t_1.00_*–*t_0.76_* and *t_0.76_*–*t_0.56_* (Figure 7). Therefore, due to the limited ranges of variation, it is possible to consider that the ski angular movements happening until 0.56 s before the landing are only adjustments for keeping the flying position stable. Therefore, in this phase, the athlete needs to adapt the ski movements to the aerodynamic changes he is subjected to. Between *t_0.56_* and *t_0.36_*, and *t_0.36_*–*t_0.16_*, the pitch movements varied by 4.4° ± 3.2° and 8.2° ± 4.8°, respectively. In particular, the angular difference of 8.2° ± 4.8° between the pitch recorded at *t_0.36_* and *t_0.16_* could be considered remarkable and related to the start of the landing preparation, considering that the angular adjustments were too wide to be related only to adaptations to the aerodynamic changes. Therefore, in line with a previous publication [4], the start of the landing preparation can be considered to happen around 0.4 s before the landing, when major movements of the hip and ankle joints were detected [4]. 

Finally, it is important to keep in mind that we calculated the ski angular range of motion (ROM) between the landing impact *t_s_* (set as reference) and specific timing before it. These timings (0.76 s, 0.56 s, 0.36 s, and 0.16 s) were chosen based on a previous publication [4]. It can be speculated that changing the timing during which the ROM of the ski movements was calculated would also change the possible correlations with impact kinetics. However, due to the variability of the ski pattern movement among athletes, defined timing before the landing was used instead of kinematic variables.

### 4.3. Limitations and Methodological Considerations 

A remarkable aspect of the study was that it was conducted on a homogeneous group of elite athletes competing at International level with ages ranging between 16 and 19 years old. The tests were performed on behalf of a scientific support for the Ski Federation during training camps. Despite the small number of tested subjects (10), the group represented the totality of the German Junior National Team. Therefore, due to the limited number of athletes belonging to the team, including in the data collection a higher number of subjects with the same technical abilities and experience was not possible. 

A limitation of the study was that the tests were carried out in two different locations, but where the ski jumping hills had a comparable size (both K-points set at 90 m) and comparable weather conditions (sunny, no wind). Moreover, two different subgroups of athletes belonging to the National Team performed the tests on the two ski jumping hills. The reason was that during the planned data collection performed within a training camp on the ski jumping hill of Oberhof, we could collect only six ski jumpers during the first day of measurements. During the second day, in fact, due to the rain and the wind, we could not carry out the tests with the remaining part of the team, because it was not possible to guarantee the same testing conditions. Therefore, we collected the data of the other four members of the National Team during the following training camp in Ramsau-am-Dachstein, on a ski jumping hill with a comparable size, always using the same combination of IMUs and force insoles. In this way, it was possible to provide the aforementioned biomechanical feedback to all the athletes of the National Team. For clarity, in the Discussion, we concentrated only on the biomechanical variables that were statistically significant on both the ski jumping hills.

Regarding the set-up, one of the main advantages was that it was not necessary to perform a calibration of the inertial sensors before doing the data collection. In fact, thanks to the algorithm proposed by Fang and colleagues [30], during the postprocessing, the raw data of the inertial sensors were reconstructed based on the design of the in-run of the ski jumping hill. The advantage of the post-initialization is very important, making the set-up easy to use, in case athletes and coaches would be interested in using the system on their own as feedback during training. In fact, not being professional researchers, they could introduce errors during the data collection. In addition, the combination of inertial sensors and force insoles can be considered relatively light (0.3 kg). Generally, the weight of the technological equipment used in the protocol is of significant importance when performing biomechanical research in sports, and it is essential in ski jumping, a sport in which the weight of the system equipment plus athlete is the main performance factor [34,35]. 

The low sampling rate of the loadsol insoles (100 Hz) could have affected the capability of measuring impact. However, publications related to this topic are discordant: Peebles and colleagues [26] highlighted under-/overestimation bias of the impact force peaks when using loadsol at 100 Hz. Other research groups did not report limitations related to the sample rate [25,27]. At the time of the data collection, loadsol insoles sampling at 200 Hz were still not available on the market. Anyway, for further studies, force insoles sampling at 200 Hz are recommendable to improve the accuracy. 

A high number of external factors (such as wind and air pressure) generally interfere with the movement of the ski jumper. Consequently, we can speculate that each jump can be considered as a specific case, also when performed by the same athlete and even though the athletes belonged to an elite level. Therefore, performing statistics is very difficult in this kind of analysis, especially when dealing with the landing that is the phase at the end of the performance and, consequently, a resultant of the previous ones [32]. As a result, the statistics performed in this study, and generally in in-field ski jumping research, need to be evaluated carefully.

## 5. Conclusions

The pitch was the main ski movement influencing the magnitude of the normal ground reaction force (GRF_max_) and the jump performance (*t_flight_*) due to its relation with the angle of attack. As a result, in order to increase the jump length and reduce the impact forces, the athlete should keep the ski more flexed during the landing preparation phase. The pitch started to considerably vary between 0.36 s and 0.16 s before the landing impact, leading to the consideration that the landing preparation started around 0.36 s before the impact.

Despite the elite level of the athletes, each subject showed an individually unique ski movement pattern during the flight phase. The analysis of the ski position could permit improving the aerodynamics of the athlete during the flight, since previous publications gave suggestions on the best ski configuration to increase the performance [1,3,6,7,32,36,37]. However, a previous study performed in a wind tunnel showed how the aerodynamics of an isolated ski depend on the combination of the roll, pitch, and yaw angle [32]. Therefore, further studies should focus on analyzing the combination of the roll, pitch, and yaw movement during in-field performance.

According to the feelings of the jumpers, the set-up constituted by the force insoles and the IMUs resulted in not interfering with the performance. Therefore, under the practical point of view, the already proven advantages of the IMUs [15,17,18,20] and the force insoles [25,26,27,28], as well as the possible advantages of their combination shown in the present study, could provide a reliable and objective feedback for coaches and athletes for monitoring the kinetics and kinematics of the ski jumping performance. To confirm this, a report with graphs about ski pitch and roll movements and the kinetics during the whole performance were provided to athletes and coaches at the end of each day of data collection. Both athletes and coaches provided a positive feedback about the report.

## Figures and Tables

**Table 1:** Average ± SD of *t_flight_*_,_ *t_landing_*, normal GRF_max_, and I of 3 jumps of 10 athletes (n = 10) who performed on the ski jumping hills of Ramsau-am-Dachstein and Oberhof.

	Ramsau-am-Dachstein (n = 4)	Oberhof (n = 6)
*t_flight_* [s]	3.33 ± 0.20	3.33 ± 0.27
*t_landing_* [s]	0.19 ± 0.03	0.16 ± 0.03
Normal GRF_max_ [BW]	3.1 ± 1.0	2.8 ± 0.8
I [BWs]	154.5 ± 33.1	146.4 ± 30.5

**Table 2:** Correlations between *t_f**light**_***_,_** *t_landing_*, normal GRF_max_, and impulse I acting on the left (L) and right (R) foot, and the ski roll, pitch, and yaw ROM of the L and R side at *t_0.16_*_,_ *t_0.36_*_,_ *t_0.56_*_,_ *t_0.76_*, and *t_1.00_* of the data collected in Oberhof (*n* = 18). The variables in bold showed a correlation between kinematics and kinetics in both the data collection of Ramsau-am-Dachstein and Oberhof.

			*t_flight_*	*t_landing_*		Normal GRF_max_		I	
				L	R	L	R	L	R
*t_0.16_*	Roll	L						*r* = 0.639**	
		R						*r* = 0.596**	
	Pitch	L						*r* = 0.492*	
		R			*r* = 0.510*	*r* = 0.520*			
	Yaw	L	*r* = 0.525*					*r* = 0.519*	
		R							
*t_0.36_*	Roll	L							
		R						***r*** = 0.618**	
	Pitch	L						*r* = −0.595*	
		R	*r* = 0.526*	*r* = 0.557*				*r* = 0.602*	
	Yaw	L							
		R	*r* = 0.504*			*r* = 0.509*		*r* = 0.629**	
*t_0.56_*	Roll	L						*r* = 0.664**	
		R						*r* = 0.517*	
	Pitch	L	***r*** = 0.714*			*r* = 0.608**		*r* = 0.685**	*r* = 0.500*
		R	***r*** = 0.499*					*r* = 0.615**	
	Yaw	L						*r* = 0.697**	
		R						*r* = 0.482*	
*t_0.76_*	Raw	L						*r* = 0.714**	
		R							
	Pitch	L	***r*** = 0.755*			*r* = 0.623**		*r* = 0.736**	
		R						*r* = 0.634**	
	Yaw	L						*r* = 0.690*	
		R						*r* = 0.478*	
*t_1.00_*	Roll	L		*r* = 0.482*		*r* = 0.494*		*r* = 0.715**	
		R							
	Pitch	L	***r*** = 0.708*			***r*** = 0.623**		*r* = 0.736**	
		R	*r* = 0.522*			*r* = 0.477 *		*r* = 0.575*	
	Yaw	L						*r* = 0.702**	
		R							

* *p* < 0.05; ** *p* < 0.005; *** *p* < 0.001

**Table 3:** Correlations between **t_flight,_** t_landing_, normal GRF_max_, and impulse I acting on the left (L) and right (R) foot, and the ski roll, pitch, and yaw ROM of the L and R side at *t_0.36_*_,_ *t_0.56_*_,_ *t_0.76_*, and *t_1.00_* of the data collected in Ramsau-am-Dachstein (*n* = 12). The variables in bold showed a correlation between kinematics and kinetics in both the data collection of Ramsau-am-Dachstein and Oberhof.

			*T_flight_*	*t_landing_*		Normal GRF_max_		I	
				L	R	L	R	L	R
*t_0.36_*	Roll	L							
		R	*r* = 0.699*					*r* = 0.712*	
	Pitch	L	*r* = 0.656*						
		R			*r* = 0.577*				
	Yaw	L							
		R							
*t_0.56_*	Roll	L							
		R							
	Pitch	L	*r* = 0.887***						
		R	*r* = 0.611*		*r* = 0.588*		*r* = 0.634*		*r* = 0.662*
	Yaw	L							
		R							
*t_0.76_*	Raw	L	*r* = −0.678*						
		R							
	Pitch	L	*r* = 0.844*						
		R	*r* = 0.660*		*r* = 0.628*	*r* = 0.611*	*r* = 0.631*		*r* = 0.715**
	Yaw	L				*r* = −0.592*			
		R							
*t_1.00_*	Roll	L	*r* = −0.736**						
		R							
	Pitch	L	*r* = 0.599*	*r* = 0.664*		*r* = 0.631*			
		R							
	Yaw	L							
		R			*r* = 0.632*				

* *p* < 0.05; ** *p* < 0.005; *** *p* < 0.001
